# Mechanism of Fluorescence Enhancement of Biosynthesized XFe_2_O_4_–BiFeO_3_ (X = Cr, Mn, Co, or Ni) Membranes

**DOI:** 10.1186/s11671-016-1747-4

**Published:** 2016-12-07

**Authors:** Liang Bian, Hai-long Li, Hai-liang Dong, Fa-qin Dong, Mian-xin Song, Li-sheng Wang, Wen-ping Hou, Lei Gao, Xiao-yan Zhang, Tian-liang Zhou, Guang-ai Sun, Xin-xi Li, Lei Xie

**Affiliations:** 1 Institute of Gem and Material Technology, Hebei GEO University, Shijiazhuang, 050000 Hebei China; 2 Key Laboratory of Solid Waste Treatment and Resource Recycle, Ministry of Education, South West University of Science and Technology, Mianyang, 621010 Sichuan China; 3 Key Laboratory of Functional Materials and Devices under Special Environments, Chinese Academy of Sciences, Urumqi, 830011 Xinjiang China; 4 Department of Geology and Environmental Earth Science, Miami University, Oxford, 45056 USA; 5 Institute of Nuclear Physics and Chemistry, CAEP, Mianyang, 621900 Sichuan China

**Keywords:** Ferrite, Heterostructures, Hematite, *Shewanella oneidensis* MR-1, Fluorescence

## Abstract

Ferrites–bismuth ferrite is an intriguing option for medical diagnostic imaging device due to its magnetoelectric and enhanced near-infrared fluorescent properties. However, the embedded XFO nanoparticles are randomly located on the BFO membranes, making implementation in devices difficult. To overcome this, we present a facile bio-approach to produce XFe_2_O_4_–BiFeO_3_ (XFO–BFO) (X = Cr, Mn, Co, or Ni) membranes using *Shewanella oneidensis* MR-1. The perovskite BFO enhances the fluorescence intensity (at 660 and 832 nm) and surface potential difference (−469 ~ 385 meV and −80 ~ 525 meV) of the embedded spinel XFO. This mechanism is attributed to the interfacial coupling of the X–Fe (e^−^ or h^+^) and O–O (h^+^) interfaces. Such a system could open up new ideas in the design of environmentally friendly fluorescent membranes.

## Background

Recently, a special focus has been set on the high-photostability photoluminescence and photothermal effect of the spinel–perovskite heterojunctions (e.g., CrFe_2_O_4_–BiFeO_3_, CoFe_2_O_4_–BiFeO_3_ [1], MnFe_2_O_4_–BiFeO_3_, NiFe_2_O_4_–BiFeO_3_ [2], etc.), providing an opportunity for the development of a new generation of narrow band-gap heterointerface devices. Of particular interest are the spinel–perovskite intercalated nanocomposites, such as XFO–BFO (X = Cr, Mn, Co, or Ni), where the fluorescent effect is ascribed to the local conduction at the vertical interface. Therein, two weak interfacial coupling peaks of nanoscale spinel are observed from the visible to near-infrared range (near 690 nm and 840 nm) [3]. The interfacial coupling strength of the Fe–O tetrahedron are enhanced due to the O–O hybridization reaction, being affected by the number of binded oxygen atoms of perovskite-type BFO membrane [4]. These so-called spinel–perovskite heterojunctions can exhibit higher interfacial coupling intensity than their laminate counterparts because of higher interfacial area between the two phases [5].

In general, it is difficult for the spinel-type XFO to coat onto perovskite-type BFO membrane directly by the effect of the lattice mismatch and randomly located magnetic pillars [6]. To solve these problems, recent efforts have thus been focused on the complex structure of perovskite–spinel membrane [7]. This was realized previously by embedding small XFO seeds into a thin BFO layer [8], either by surface drilling or prepatterning [9], which guided the growth of the composite. In those previous articles [10], the p–n characterization of the templated nanocomposites was limited to macroscopical measurements at the remanence and interfacial coupling [11], ignoring the enhanced fluorescence at visible and near-infrared range that the few scattering centers can absorb most of the solar spectra.

In this case, we presented a biosynthetic approach to produce uniform pores on the BFO membrane and inserted the iron-based spinel into the pores. We succeeded in enhancing the fluorescence data to near-infrared range and finally demonstrated how relevant the fluorescence varies with XFO–BFO heterostructures. Our findings open new pathway to design the functional nanostructures.

## Methods

*Shewanella oneidensis* MR-1 and BFO membranes were cultured and synthesized in a chemically defined minimal medium as described previously [12]. The medium including 90 mM X-modified FeOOH and 2.3 × 10^6^ cells were 100 ml. All treatment tubes were incubated in the dark at 30 °C in 30 days until the end of the experiment. The XFO–BFO membranes were finally obtained after freeze-drying.

Scanning electron microscopy (SEM) and energy-dispersive X-ray spectroscopy (EDS) of XFO–BFO membranes were performed on a Cu plate using a Hitachi S-4800 field emission machine [10]. The existence of X^2+^ and Fe^3+/2+^ ions was tested by X-ray photoelectron spectroscopy (XPS) in a Krotos-XSAM 800 multifunctional electron spectrometer using a monochromatic Al Kα radiation. The anode was operated at 12 kV and 20 mA. The spectrometer was equipped with a DS300 unit for data acquisition [13]. X-ray diffraction data were collected using an X-ray diffractometer (BRUKE D8 ADVANCE, Germany) with Cu Ka radiation (*k* = 0.154 nm) with a working voltage of 40 kV and a current of 40 mA at a step of 0.02 in the range of 2θ = 5 ~ 90°. The Raman scattering measurements (Labram HR evolution, Horiba scientific, France) were conducted at room temperature under a backscattering geometric configuration using a WITec-Alpha confocal micro-Raman system [14].

Photoluminescence (PL) emissions of XFO–BFO membranes at room temperature were obtained at 300–550 and 420–900 nm with an excitation wavelength at 250 and 550 nm, respectively. The slit widths of excitation and emission were 5.0 nm (USB 4000, Ocean Optics, USA). The corresponding fluorescent microscopic images were obtained at 405, 488, and 635 nm using an inverted fluorescence microscope (Olympus, Southend-on-Sea, UK) [15]. The measurements of surface potentials and polarization angles were performed using an atomic force microscopy (AFM) and Kelvin probe force microscopy (KPFM) on an atomic force microscope (Asylum Research MFP-3D) [13]. The electron transfer mechanism was simulated via the density functional theory (DFT) + *U* method based on the generalized gradient corrected Perdew–Burke–Ernzerhof functional (GGA–PBE) using density functional theory (Castep, Materials studio, Accelrys, USA) [16]. The Coulomb and screened exchange parameters (*U*, *J*) were set to 5 and 1 eV, respectively [17]. The electron wave functions at each *k*-point were expanded [18]. A kinetic energy cutoff of 300 eV for the electrons was used well within the convergence of a total energy calculation. A 2 × 2 × 2 super cell was introduced for the interstitial plane wave as was a 5 × 5 × 5 *k*-point mesh for integration over the Brillouin zone [19].

## Results and Discussion

### Characterization of XFO–BFO Membranes

**Fig. 1:**
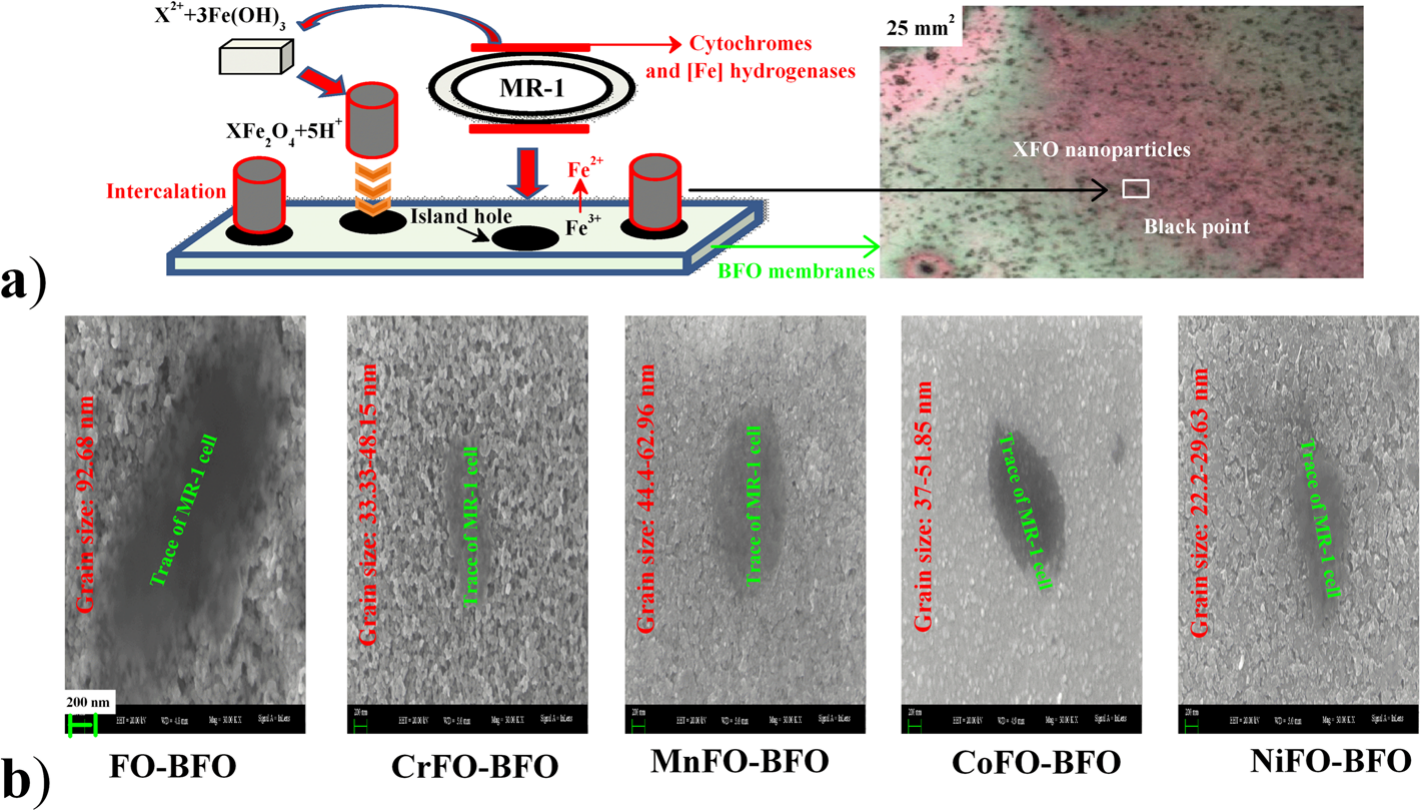
**a** Illustration of synthesis process of XFO–BFO membrane and its microscope picture (5 × 5 mm^2^). During the synthesis process, the first step is that *Shewanella oneidensis* MR-1 possesses a periplasmic [Fe]-hydrogenase that drives the microbial reduction of Fe^3+^ [Fe(OH)_3_: cuboid] and the release of Fe^2+^ ions to the aqueous solution. The second step is that the =Fe^2+^(OH)_2_ surfaces can absorb and immobilize X^2+^. The third step is that the functional =Fe^2+^(OH)_2_ groups provide high surface area and functional sites which allow the chemical complexation of X^2+^ [21, 22]. **b** SEM images (200 nm) of XFO–BFO membranes

The BFO matrix grown on Pt forms uniform plateaus. In Burns’ opinion [20], the extracellular polymeric substances cytochrome and [Fe] hydrogenases outside *S. oneidensis* MR-1 can reduce Fe^3+^ to Fe^2+^ on the surface of BFO membrane for creating an island hole to absorb the XFO. Besides, the FeOOH is reduced to spinel-type Fe_3_O_4_, as shown in Fig. 1a. The reduced Fe^2+^ ion plays an important role in compensating for the charge imbalance and also mediates the electron transfer between MR-1 and X^2+^. In these processes, the microbial reduction of X^2+^ loaded poly Fe(NO)_3_ by *S. oneidensis* MR-1 are considered involving three key steps [21]. These processes have been well documented in the literature [22] according to the following three equations: (1) Fe_2_O_3_ + 3H_2_O → 2Fe^3+^(OH)_3_ (cuboid) → 2 = Fe^2+^(OH)_2_ + 2H^+^; (2) X^2+^ + = Fe^2+^(OH)_2_ → X^2+^–OH–Fe^2+^–OH; (3) X^2+^ + = Fe^2+^(OH)_2_ → X^2+^OFe^2+^O + 2H^+^, where Eq. (1) describes the reduction reaction of Fe_2_O_3_ by *S. oneidensis* MR-1 in a water environment. Equation (2) reflects the electrostatic adsorption of X^2+^ on the tetrahedral = Fe^2+^(OH)_2_ surfaces at the acidic condition. Equation (3) illustrates the chemical complexation of X^2+^ with the functional = Fe^2+^(OH)_2_ site. Finally, the X^2+^-doped tetrahedral = Fe^2+^(OH)_2_ groups lost H_2_O molecules to form a magnetite structure (cylinder).

Near the area of MR-1 cells on the surface of the BFO membrane, the periplasmic [Fe]-hydrogenase is equally sensitive to surface Fe^3+^ ions, releasing a part of Fe^2+^ ions. The dissociated Fe^2+^ sites, as the island holes (black circular), can absorb the XFO particles (see Fig. 1b). The intercalated XFO particles as the growth centers show high surface waviness, and the other small XFO particles did not grow up to the top surface and were overgrown with BFO membrane [6]. The small spherical XFO intercalates into the island hole, forming the XFO–BFO membrane. It is very clear that the XFO nanoparticles are in uniformity near the trace of MR-1 cell on the BFO membranes (see Fig. 1b). When X was doped into FO particles, the calculated formation enthalpies increase from −34690.42 eV (FO) to −47503.09 (CrFO), −33000.22 (MnFO), −36106.85 (CoFO) and −38601.21 (NiFO) eV, respectively. Under the same synthesis process, the XFeO crystal lattices need more energies to grow the same size compared to that of FO; therefore, the XFO nanoparticles are distributed as a narrow size range of 22.2–62.96 nm, comparing to that of FO nanoparticle (92.68 nm). It should be noted that the obvious agglomeration between surface magnetic MnFe_2_O_4_ and NiFe_2_O_4_ nanoparticles will inevitably occur in the growth process, with the particles freely growing into the density sheets [23].

**Fig. 2:**
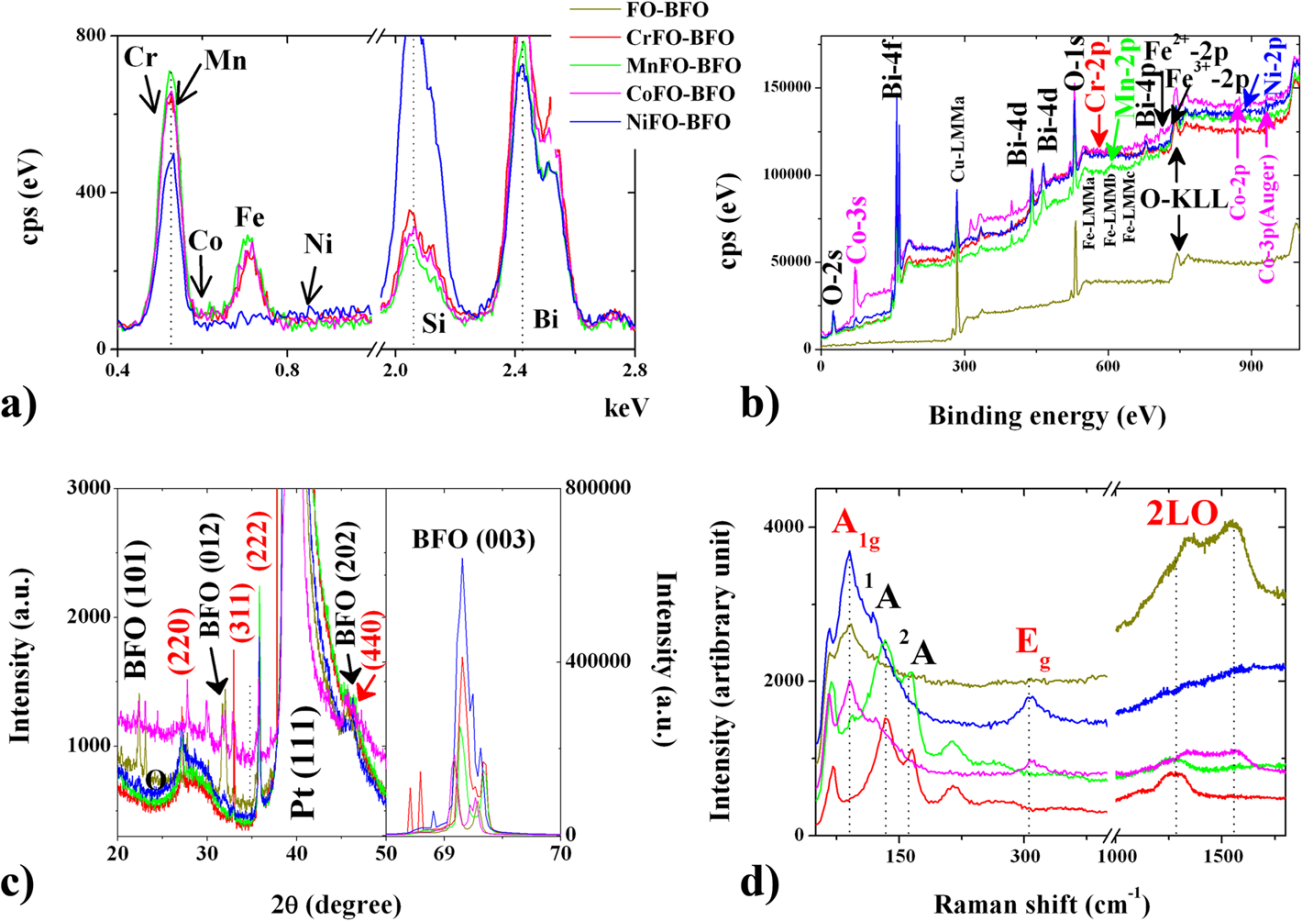
**a** SEM–EDS, **b** XPS, **c** XRD and **d** Raman curves of XFO–BFO membranes. The curves are FO–BFO (*yellow*), CrFO–BFO (*red*), MnFO–BFO (*green*), CoFO–BFO (*violet*), and NiFO–BFO (*blue*)

SEM–EDS spectra in Fig. 2a confirms the co-existence of Fe, X, Bi, and O elements. The rates of cation atom contents (at.%) are 1.26:25.85:23.59 (Cr:Fe:Bi), 0.9:11.59:22.66 (Mn:Fe:Bi), 0.4:23.98:16.25 (Co:Fe:Bi), and 0.05:19.59:19.31 (Ni:Fe:Bi), respectively. The results show that X^2+^ ions occupy a part of tetrahedral Fe^2+^ sites. To identify the chemical bonding in these membranes, the valence of Fe in the thin films was studied by XPS, as displayed in Fig. 2b. The 2p^1/2^ and 2p^3/2^ spin-orbit doublet components of the Fe photoelectrons are located at near 713.5 and 724.5 eV, respectively. The fitting analysis of Fe 2p^3/2^ shows the Fe^2+^ and Fe^3+^ ions coexisting in the XFO–BFO membranes, being sensitive to the oxygen vacancy (O-KLL, the electron shell hole at approximately 730 eV). An interesting small peaks are located at the low-energy area (Cr^2+^−2p: 594 eV; Mn^2+^–2p: 660 eV) and high-energy area (Co^2+^–2p: 809 eV; Ni^2+^–2p: 888 eV) near the Fe peaks and oxygen vacancy, respectively. It is believed that the reduced Fe^3+^ and low (or high) energy X^2+^ ions imply that the formation of oxygen vacancy will be greatly promoted (Cr^2+^–2p; Mn^2+^–2p) or suppressed (Co^2+^–2p; Ni^2+^–2p) for the Fe^3+^–O^2−^–Fe^2+^ charge compensation. The results hence inferred that the BFO-rich and XFO-rich microregion causes the difference of surface potential.

To verify the crystalline structure of the as-synthesized sample, typical power XRD patterns are observed as shown in Fig. 2c. The clear diffraction peaks at 2θ angles (at approximately 22.5°, 31°, 46°, 28°, 31.5°, 36°, and 46.5°) and can be assigned to the (101), (012), and (202) characteristic reflections of BFO (JCPDS 20-0169) and the (220), (311), (222), and (440) characteristic reflections of the cubic structure of magnetite XFO (JCPDS 19-0629) [4, 8, 10]. The narrow and sharp peaks suggest that there is a highly crystalline nature (average crystalline sizes 4.5–5 nm) for the obtained XFO–BFO heterostructure. Moreover, the distinct characteristic Raman modes in Fig. 2d can be used to distinguish the interactions of XFO–BFO. Focusing on the peaks at 146–152 cm^−1^, it represents the non-degenerate (^1^A and ^2^A) mode of the BFO membrane. The blue shift (85 cm^−1^) of the XFO Raman peak could be attributed to the compressive strain from the lattice mismatch between the XFO nanoparticles and BFO membranes. Dominant Raman band at 301 cm^−1^ corresponds to twofold degenerate (E_g_) mode of the XFO normal spinel with X^2+^ and Fe^2+^ ions at the tetrahedral sites. Typically, a broad asymmetric peak at around 1370–1560 cm^−1^ is attributed to the second-order (2LO) Raman mode of the structural defects on the spinel–perovskite grain boundary. This special optical property reduces the phonon interaction, reflecting the existence of oxygen vacancy in the interfaces.

### Fluorescence Enhancement of XFO–BFO Membranes

Photoluminescence (PL) emission spectra are useful for disclosing the transfer and recombination processes of the photo-generated electron hole pairs [24]. Here, the main emission peaks at 340–431 nm when the material is excited by 250 nm Xe light correspond to the intrinsic band-gap emission of tetrahedral Fe^2+^–O^2−^ orbital, and the octahedral Fe^3+^–O^2−^ orbital has a broad green emission peak at 449–496 nm. In the interface, the quantum wells are formed in the interface between BFO and XFO, by 525 nm Xe light exciting. The dominant acceptor (Fe–O bond of XFO) serves as the main d-orbital defect, increasing in the density of acceptor state by capturing one electron of O^2−^ anions (BFO). The orbital hybridization at the tetrahedral XFe–O interfacial coupling creates two weak quantum wells between BFO and XFO interfaces. As a result, the recombination of the electrons being trapped in the valence band might be responsible for the existence of two peaks at 660 nm and 832 nm [3].

**Fig. 3:**
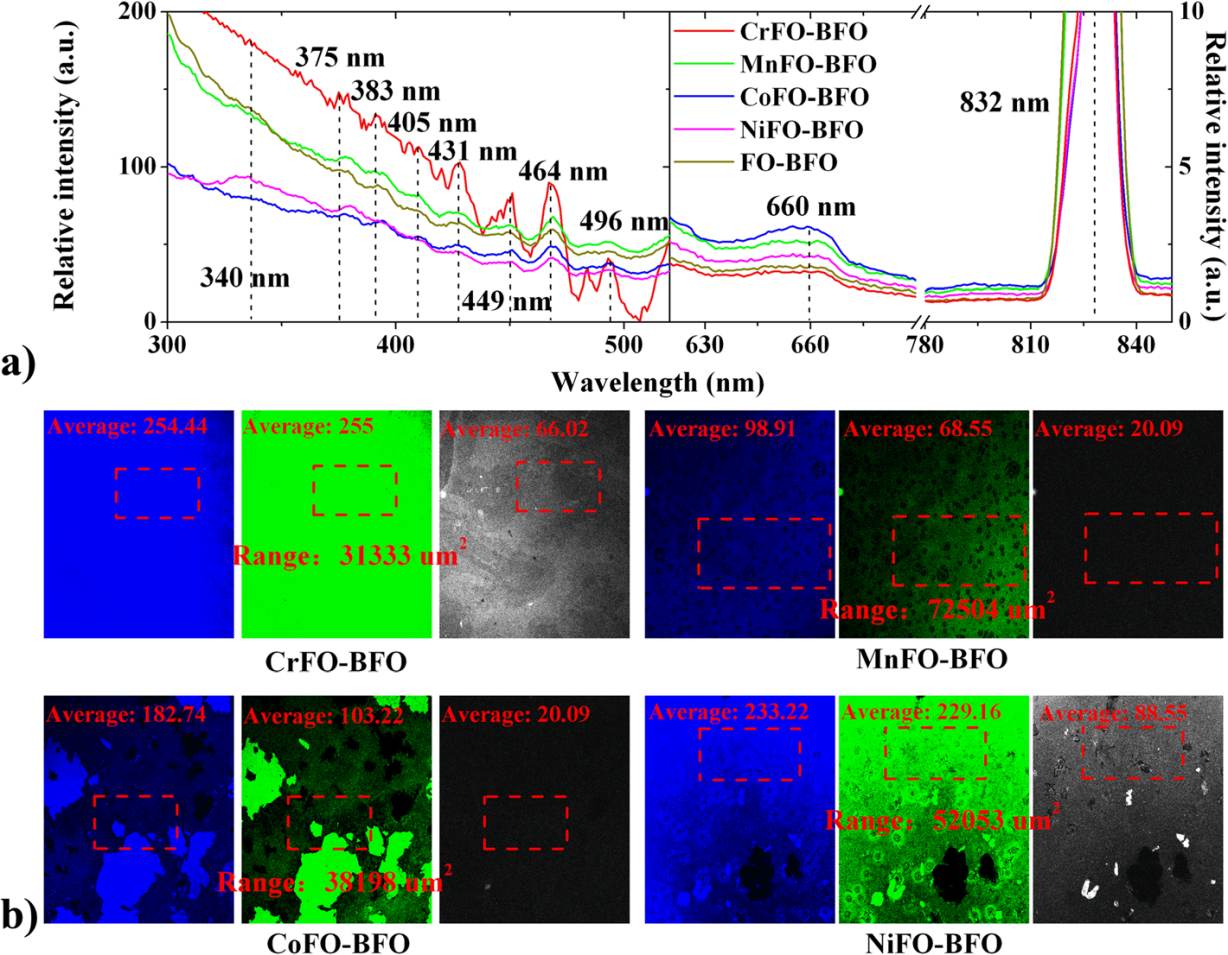
**a** Fluorescence spectra and **b** images of XFO–BFO membranes. Therein, the excitation wavelength of **a** fluorescence spectra are 250 (*left*) and 550 (*right*) nm, respectively. The main emission peaks at 340–431 nm (*green*) and 449–496 nm (*green* and *blue*) correspond to the *intrinsic blue* and *green* emission of octahedral Fe^3+^–O^2−^ (XFO and BFO) orbitals and tetrahedral Fe^2+^–O^2−^ (XFO) orbitals, respectively. The excitation wavelengths of **b** fluorescence microscopies are 405 (*green*), 488 (*blue*), and 635 (*gray*) nm

Furthermore, a wide light-emitting region in Fig. 3b predicts that there is an enhanced electron transfer density of tetrahedral XFe–O bond due to the presence of a weak O–O interface, which may be the sources of dissipation of light in the form of absorption or scattering. The corresponding photofluorescence intensity appears in the visible gray region (832 nm), being higher than the literature values (375–625 nm) [25, 26]. However, it should be noted that the oxygen vacancy captures a part of active electron in surface X–O–Fe. The tetrahedral X–Fe electron state density and its spin quantum number is weakened (see Section “Mechanism of fluorescence enhancement”), improving simultaneously the surface adsorption. It makes these membranes promising from both adsorption and detection point of view.

### Mechanism of Fluorescence Enhancement

**Fig. 4:**
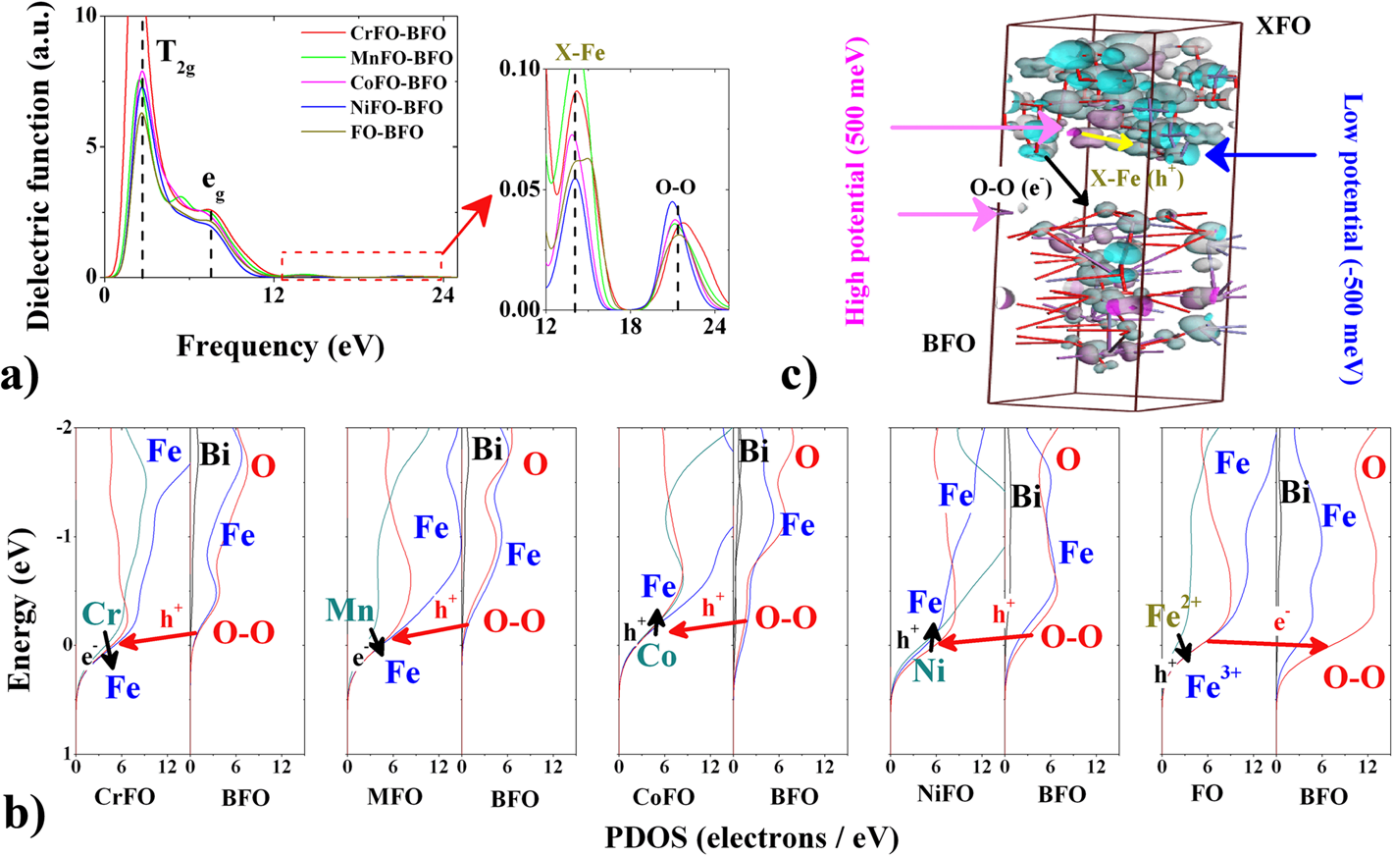
**a** Dielectric functions, **b** PDOSs, and **c** surface potentials of XFO–BFO membranes by DFT simulation. Therein, the scissors were used to make rigid upward shift of conductive bands by 2.5 eV (BFO) and 2 eV (XFO) [19]

The inner electronic transitions of XFO–BFO interfaces are calculated by the dielectric functions (see Fig. 4a). The peak T_2g_ (2.8 eV) corresponds mainly to the electronic transition from octahedral O–2p valence band to Fe-3d high-energy conduction band in BFO (or X–p (or d) in XFO) [27]. The peak e_g_ (6.8 eV) is ascribed to the electronic transition from O–2p to X–2p in tetrahedron of XFO. Here, it should be noted that the O–O hybridization in the interface enhances two weak interfacial coupling peaks of nanoscale spinel at near-infrared range, due to that two-dimensional electron gases exist at the (i) X–Fe (e^−^ or h^+^) and (ii) O–O (h^+^) interfaces [28]. The Mulliken charges confirm that two small peaks (frequency, 14 eV and 21 eV) correspond mainly to the X–Fe and O–O interfaces. In the tetrahedral X–Fe–O interface, the Fe^2+^ (Cr^2+^ or Mn^2+^; Co^2+^ or Ni^2+^) as electron donor provides or captures a part of electron (0.11 *e* or 0.02 *e*; 0.06 *e* or 0.35 *e*) to Fe^3+^ electron acceptor, promoting or suppressing Fe^3+^–O^2−^–Fe^2+^ charge compensation. The order of electron transfer quantities directly corresponds to the order of average intensities of photofluorescence images.

**Fig. 5:**
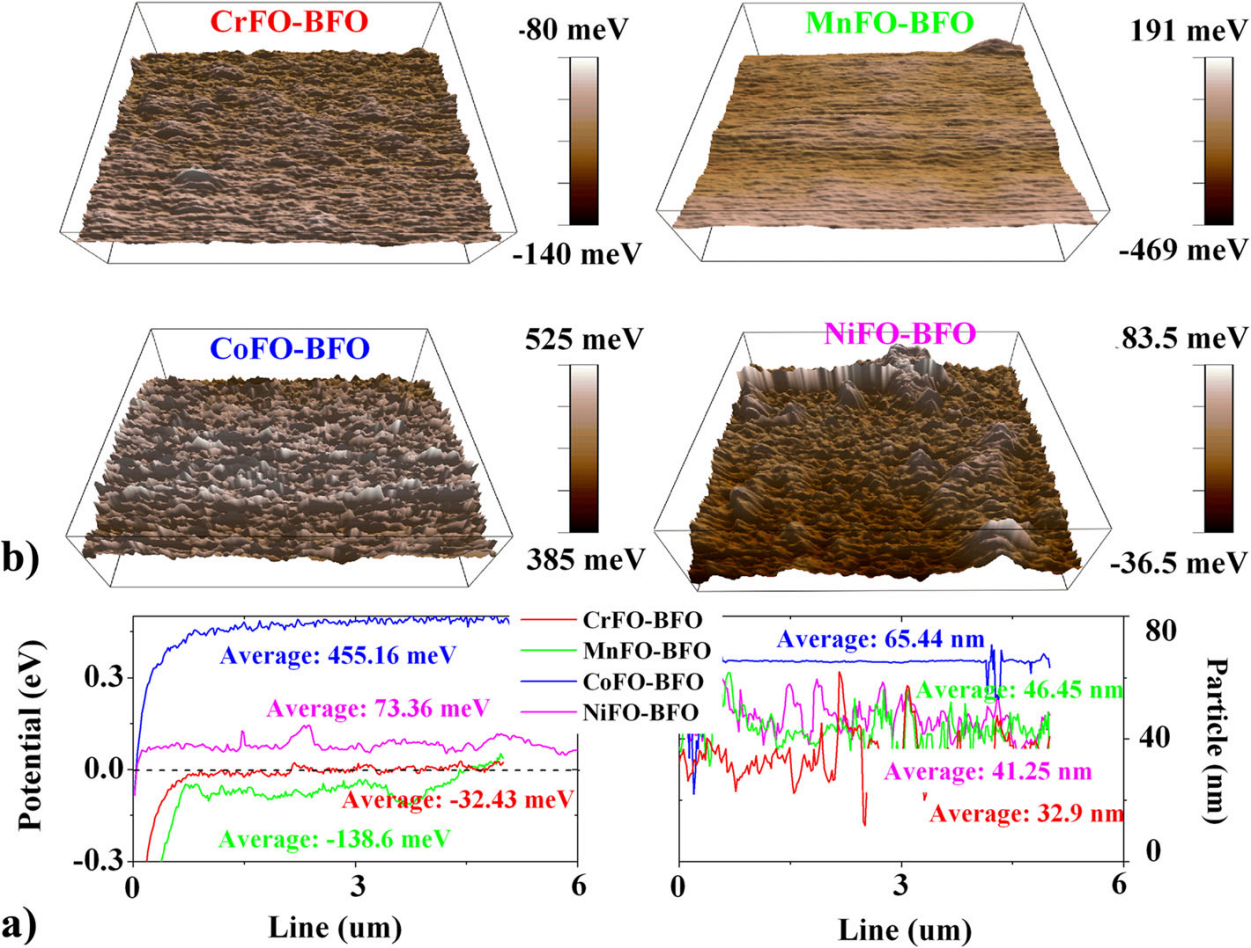
**a** Surface potential images and **b** the relative potential curves of XFO–BFO membranes by AFM–KPFM measurement. Therein, the scanning area is 5 μm × 5 μm. The particles are essentially in agreement with the differences of grain sizes in SEM images

Ultimately, the weakened O electron gas (−0.7 *e* → −0.63~ −0.62 *e*) in XFO plays an important role on determining the change of electron transfer behavior (*e* ^−^ → h^+^ → h^+^ → *e* ^−^ for XFO → BFO) [29, 30]. It appears at the top of valence band in Fig. 4b, therein, long-range Fe↓–O↑ d*–p orbital hybridization at Fe–O–Fe interface enhances the surface potential at a small area. The O (BFO) up–spin (↑) electron preferentially transfers into the orbital of O↑ (XFO), due to enhanced O–O p–p energy barrier (Δ = 0.2−0.5 eV) near Fermi point. It weakens the O–Fe p–d orbital strength (because of the reduced PDOS data near Fermi point) in the tetrahedron of XFO, and therefore, the interfacial coupling weakens the anti-bonding orbital (d*) potential of Fe down-spin (↓). The tetrahedral Fe–O fluorescence intensity is enhanced, and it appears simultaneously with surface potential transition. Figure 5a indicates that the average potentials are enhanced (73.36 meV and 455.16 meV) or weakened (−32.43 meV and −138.6 meV) comparing to that (7 meV) of FO–BFO. They are uniform (error <50 meV; FO–BFO 54 meV) although the surface particles show uneven heights (<30 nm) at the 5-μm slash region. Therein, the relative interfacial coupling activity is distinguished according to the energy difference between X and Fe ions (Cr –2457.31 eV, Mn –644.37 eV, Fe −855.87 eV, Co −1034.68 eV, Ni −1347.04 eV). Besides, the annihilated effective electron is confirmed on the X–Fe or O–O interfaces, whereas the surface potentials split into low surface potential (–500 meV) and high surface potential (500 meV) (see Fig. 4c). These are in accord with the surface potential splitting of AFM–KPFM experimental phenomenon (high potential: dark area; low potential: light area). The difference of average potential data between high potential and low potential is 60–660 meV, as displayed in Fig. 5b. It is ascribed to the facts that the BFO surrounded by XFO, where the potential difference (Sdev: 15–20 meV) may be caused by the XFO-rich and BFO-rich in the small area.

## Conclusions

In conclusion, a novel bio-induced phase transition method for the growth of highly ordered enhanced fluorescence of spinel XFO embedded in perovskite BFO membrane is proposed here. We described the interfacial coupling mechanism for good enhanced fluorescent functionality of the present XFO-BFO membranes, using AFM-KPFM, PL, and DFT measurements. The present work demonstrates that the X-doping changes the electron transfer behavior from electron transfer to hole transfer in the XFO-BFO interface at 660 nm and 832 nm. The long-range O–O p–p and X–Fe p–d orbital hybridization verifies the difference of surface potential (−469 ~ 385 meV and −80 ~ 525 meV), being effective by the energy difference of X and Fe. Therefore, the bio-induced method provides a novel method for most of the systems based on Fe_2_O_3_–FeFe_2_O_4_ phase transition in the enhanced fluorescence spinel–perovskite systems.

The current study provides new information regarding the enhanced fluorescence complex heterostructures, for improving the response sensitivity and uniform property in the fluorescence detection. Further investigation will be focused on the cycled magnetic (or infrared) excitated electron transfer processes in the water enviroment to garner a better understanding of the fast-response fluorescence process.
